# Long noncoding RNA LINC00511 contributes to breast cancer tumourigenesis and stemness by inducing the miR-185-3p/E2F1/Nanog axis

**DOI:** 10.1186/s13046-018-0945-6

**Published:** 2018-11-27

**Authors:** Guanming Lu, Yueyong Li, Yanfei Ma, Jinlan Lu, Yongcheng Chen, Qiulan Jiang, Qiang Qin, Lifeng Zhao, Qianfang Huang, Zhizhai Luo, Shiqing Huang, Zhongheng Wei

**Affiliations:** 1 Department of Mammary and Thyroid Gland Surgery, Youjiang Medical College Affiliated Hospital, Baise, 533000 Guangxi China; 2 The First Affiliated Hospital of Jinan university, Huangpu Road, No. 613, Guangzhou, 510630 Guangdong China; 3 Department of Oncology, Youjiang Medical College Affiliated Hospital, Baise, 533000 Guangxi China; 4 Department of Dental, Youjiang Medical College Affiliated Hospital, Baise, 533000 Guangxi China; 5 Department of Tumor, Youjiang Medical College Affiliated Hospital, Zhongshan Second Road, No. 18, Baise, 533000 Guangxi China

**Keywords:** Breast cancer stem cells, LINC00511, E2F1, Nanog, miR-185-3p

## Abstract

**Background:**

Emerging evidence have illustrated the vital role of long noncoding RNAs (lncRNAs) long intergenic non-protein coding RNA 00511 (LINC00511) on the human cancer progression and tumorigenesis. However, the role of LINC00511 in breast cancer tumourigenesis is still unknown. This research puts emphasis on the function of LINC00511 on the breast cancer tumourigenesis and stemness, and investigates the in-depth mechanism.

**Methods:**

The lncRNA and RNA expression were measured using RT-PCR. Protein levels were measured using western blotting analysis. CCK-8, colony formation assays and transwell assay were performed to evaluate the cell proliferation ability and invasion. Sphere-formation assay was also performed for the stemness. Bioinformatic analysis, chromatin immunoprecipitation (ChIP) and luciferase reporter assays were carried to confirm the molecular binding.

**Results:**

LINC00511 was measured to be highly expressed in the breast cancer specimens and the high-expression was correlated with the poor prognosis. Functionally, the gain and loss-of-functional experiments revealed that LINC00511 promoted the proliferation, sphere-formation ability, stem factors (Oct4, Nanog, SOX2) expression and tumor growth in breast cancer cells. Mechanically, LINC00511 functioned as competing endogenous RNA (ceRNA) for miR-185-3p to positively recover E2F1 protein. Furthermore, transcription factor E2F1 bind with the promoter region of Nanog gene to promote it transcription.

**Conclusion:**

In conclusion, our data concludes that LINC00511/miR-185-3p/E2F1/Nanog axis facilitates the breast cancer stemness and tumorigenesis, providing a vital insight for them.

**Electronic supplementary material:**

The online version of this article (10.1186/s13046-018-0945-6) contains supplementary material, which is available to authorized users.

## Introduction

Breast cancer is one of the most common cancers in women worldwide and is the leading cause of cancer-related death in women [1–3]. The primary method for treating breast cancer is surgery, chemotherapy, and/or radiation therapy, greatly improving the therapeutic effects [4]. Research has shown that stem cell characteristics play a role in the recurrence and regeneration of breast cancer [5]. A special subgroup of tumor cells exists in the tumor, called cancer stem cells (CSCs) or tumor-initiating cells. CSCs have the potential of self-renewal, multidirectional differentiation, infinite proliferation and tumor reconstruction [6]. They are regarded as the source of tumor development, differentiation, invasion and metastasis, radiochemotherapy resistance and recurrence [7]. Breast cancer stem cells (BCSCs) have been verified to be the vital promoting factor for the breast cancer cells proliferation and self-renewal ability [8].

Long noncoding RNAs (lncRNAs) play a role in epigenetic regulation in human pathophysiology [9–11] and a large have been found to participate in cancer tumorigenesis [12]. For example, FEZF1-AS1 modulates Nanog expression through sponging miR-30a, suggesting the regulation of FEZF1-AS1/miR-30a/Nanog [13]. Further, H19 acts as a competitive endogenous RNA for miRNA let-7 resulting in the release of hypoxia-inducible factor 1α (HIF-1α), leading to increased PDK1 expression, thereby regulating the cancer stem-like characteristics [14].

Long intergenic noncoding RNA 00511 (LINC00511) is an oncogene that influences tumor size, metastasis, and poor prognosis. LINC00511 binds histone methyltransferase EZH2 and specifies the histone modification pattern on p57 [15]. It also acts as an oncogene in squamous cell carcinoma and pancreatic ductal adenocarcinoma [16, 17]. In the pre-experiments, we have performed large scale screening for the differentially expressed lncRNAs for breast cancer. After filtration and consultation, we select the LINC00511 as the target.

In the present study, LINC00511 acts as an oncogenic RNA in breast cancer tumorigenesis. LINC00511 is located in the cytoplasm and functions as a miR-185-3p ‘sponge’ and targets E2F1. The transcription factor E2F1 binds with the promoter of Nanog to activate its expression at the transcriptional level. The LINC00511/miR-185-3p/E2F1/Nanog axis may have therapeutic potential for breast cancer stemness and tumorigenesis.

## Materials and methods

### Clinical subjects and specimens

A total of 39 cases of breast cancer subject specimens were recruited at Youjiang Medical College Affiliated Hospital. The tumor tissue was excised during surgery and a pathological classification was made and graded by two independent experienced pathologists. All participators provided informed consent and the procedures were approved by the Ethics Committee of the Youjiang Medical College Affiliated Hospital.

### Cell lines and culture

All the cell lines were provided by the American Type Culture Collection (ATCC), including normal human breast epithelial cell (MCF-10A) and breast cancer cells (MDA-MB-468, MDA-MB-231, MDA-MB-453, MCF-7). Cells were cultured in Dulbecco’s modified Eagle’s medium (DMEM; Gibco) and supplemented with 10% fetal bovine serum (FBS) in atmosphere containing 5% CO_2_ at 37 °C.

### Cell transfection

The miR-185-3p inhibitor, E2F1 shRNA, and negative control (NC) were purchased from Gene-Pharma Cells Company. The LINC00511 and E2F1 sequence were sub-cloned into the pcDNA3.1 vector (Invitrogen) to enhance LINC00511 expression (pcDNA-LINC00511) and empty pcDNA3.1 vector (pcDNA-NC). The transfection was performed using Lipofectamine 2000 (Invitrogen, Carlsbad, Calif, USA) according to the manufacturer’s instructions. Short hairpin RNA targeting LINC00511 (shRNA-LINC00511) and control shRNA (shRNA-NC) were provided by Santa Cruz Biotechnology (Santa Cruz, CA, USA). The shRNA sequences used in the transfection are presented in Additional file 1: Table S1.

### RNA isolation and quantitative polymerase chain reaction

Total RNA was isolated from breast cancer tissue and cells using Direct-zol™ RNA kits (Zymo research, USA) following the manufacturer’s protocol. RNA concentration was checked using a NanoDrop 2000 spectrophotometer (Termo scientifc, USA). cDNA was produced and the reverse transcription (RT) reaction was done using PrimeScrip-RT Reagent Kit (Takara) and ABI7900 system (Applied Biosystems, USA). The relative gene expression (fold change) was calculated using the 2^-ΔΔCt^ method. The primer sequences used in the PCR are presented in Additional file 2: Table S2.

### Sphere-formation assay

Breast cancer cells MDA-MB-231, and MCF-7 that transfected with shRNAs or plasmids were seeded in the six-well culture plates (Corning, NY, USA). Cells (2 × 10^5^) and were cultured in the serum-free DMEM medium with EGF, hFGF (Peprotech, USA), insulin, and penicillin/streptomycin (Gibco) added as previously described [18]. Spheroids clones were fixed and stained with crystal violet, and counted under light stereomicroscope (Olympus, Tokyo, Japan).

### Proliferation CCK-8 and colony formation assay

CCK-8 assay was conducted using a CCK-8 assay kit (Dojindo Japan). The transfected cells were seeded into culture plates and the incubated cells were treated with 10 μl of CCK-8 reagent. Absorbance was measured at 450 nm.

### Transwell invasion assays

The breast cancer cells were seeded on the member pre-coated with matrigel (BD Biosciences, San Jose, CA, USA) using 24-well transwell chamber (Corning). After 24 h of incubation, the cells on the upper surfaces were scraped, and the invaded cells were fixed with 4% paraformaldehyde and stained with Giemsa. Cells were then counted under a light microscope.

### Western blot assay

Western blot was conducted as previously described [19]. The proteins were extracted from the breast cancer specimens using RIPA lysis buffer (Beyotime, Shanghai, China). BCA Protein Assay Kit was used to evaluate the protein concentration. The protein was separated from the sample buffer using SDS-PAGE, transferred into PVDF membranes and blocked with 5% skim milk for 1 h. The primary antibodies were provided by Abcam Company, including anti-Nanog (ab109250, 1:1000), anti-Oct4 (ab184665, 1:1000), anti-SOX2 (ab137385, 1:1000). The member was incubated at 4 °C overnight and then incubated with horseradish peroxidase-conjugated secondary antibodies. Immunoblots were visualized by ECL detection system (Pierce, Rockford, IL, USA).

### Luciferase gene reporter assay

Luciferase reporter vectors containing the wild-type or mutant sequences towards the E2F1 binding of Nanog promoter region were constructed. The vectors were co-transfected with E2F1 into MDA-MB-231 cells by the Lipofectamine 2000 reagent (Thermo Fisher, USA). The activity of Renilla plasmid (Promega) was measured using Dual-Luciferase Reporter Assay Kit (Promega).

### Chromatin immunoprecipitation (ChIP)

The ChIP assay was done using the EZ-ChIP™ Chromatin immunoprecipitation kit (Millipore, USA). 1 × 10^7^ cells were incubated using anti-E2F1 (5 μg) antibody and complexes were digested. Finally, the purified DNA was measured using qRT-PCR for each Nanog promoter region site.

### Xenograft in vivo analysis

The xenograft mice in vivo assays were done according to the institutional guidelines and approved by the Animal Ethics Committee of Youjiang Medical College Affiliated Hospital. Four-week-old male null mice (about 20 g) were purchased from Shanghai SLAC Laboratory Animal Co., Ltd., (Shanghai, China). Breast cancer cells that transfected with lentivirus (sh-NC or sh-LINC00511, 5 × 10^7^ cells/ml, 0.1 ml) were injected subcutaneously. The tumor dimensions were measured every 3 days and the volume was calculated using formula π/6 (length×width^2^). After 3 weeks, mice were sacrificed by cervical dislocation and the neoplasm was excised for weighting.

### Statistical analysis

Data are presented as mean ± SD and all experiments were performed in triplicate. The difference within groups was assessed using one-way analysis of variance and independent samples t-tests. Analysis was performed using SPSS software and graphed using GraphPad Prism software. *P* value less than 0.05 was considered significant.

## Results

### LncRNA LINC00511 is ectopically over-expressed in the breast cancer tissues and cells compared to normal tissue and cells

**Fig. 1:**
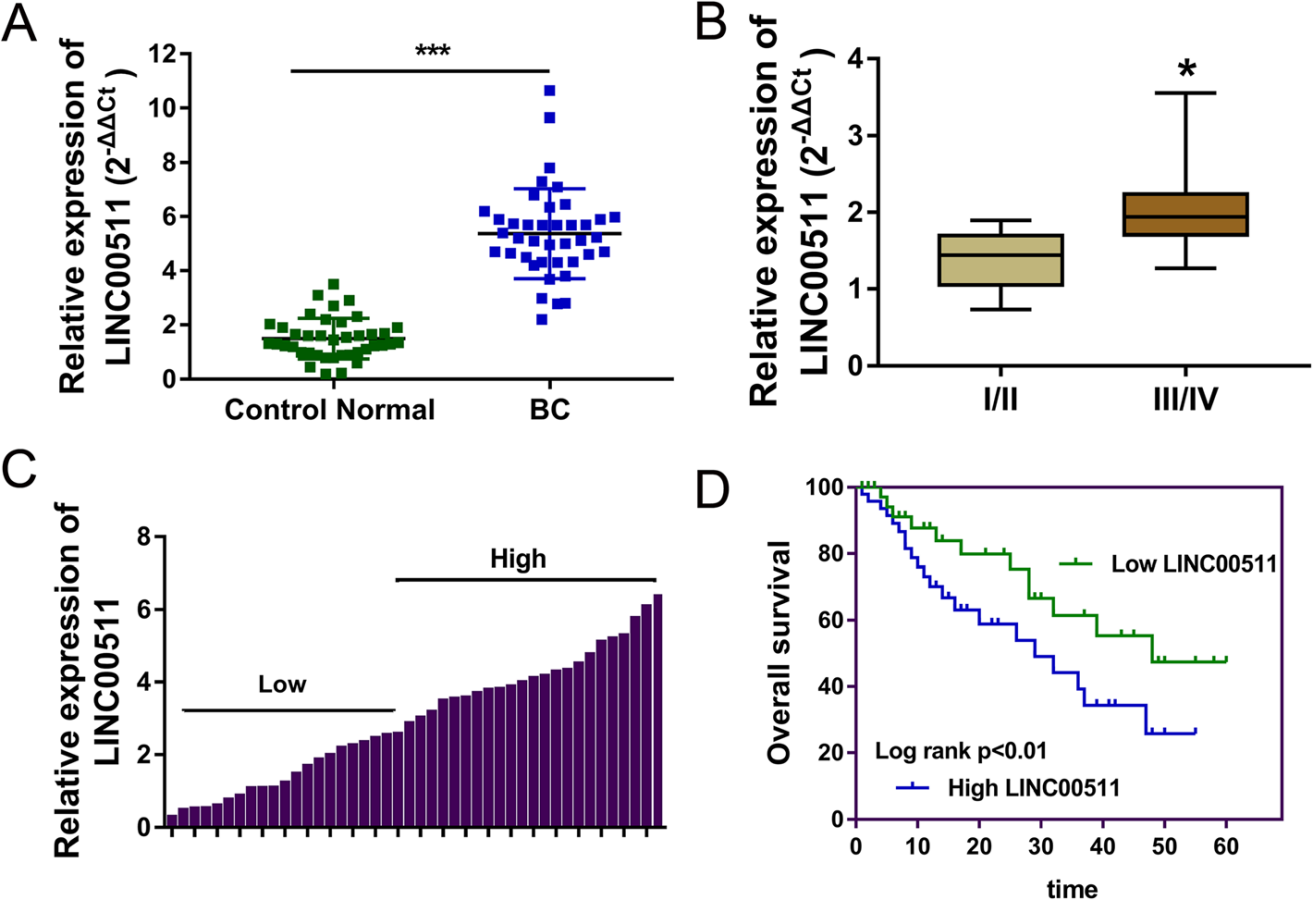
LncRNA LINC00511 is ectopically over-expressed in breast cancer tissue compared to normal tissue. **a** LINC00511 expression in the breast cancer tissue compared with normal adjacent tissue. **b** LINC00511 expression in the breast cancer tissue with TNM stage. **c** High and low expression groups according to the mean value. **d** Survival rate of patients with high or low LINC00511 expression. *** indicates *p* value less than 0.001. * indicates *p* value less than 0.05

After comparison and screening, LINC00511 was found to be significantly up-regulated in the breast cancer tissues (Table 1). In the clinical samples, LINC00511 was over-expressed in the breast cancer tissue compared to normal adjacent tissue (Fig. 1a). There was a positive correlation between LINC00511 expression and clinicopathological parameters (TNM stages), with increased expression at more advanced stage of disease\ (Fig. 1b). The cohort of breast cancer patients was divided into high and low expression groups according to the mean value or the cutoff (Fig. 1c) and Kaplan-Meier analysis and log rank analysis were used to evaluate survival rate. Data illustrated that the patients with higher LINC00511 expression had poor prognosis compared to those with lower LINC00511 expression (Fig. 1d). These results suggest that lncRNA LINC00511 is ectopically over-expressed in breast cancer tissue and cells compared to normal tissue and cells, which means LINC00511 may be an oncogene for breast cancer.

**Table 1:** The correlation within the LINC00511 expression and the breast cancer patients’ characteristics

	N	LINC00511		*P* value
		Low = 16	High = 23	
Age				
< 50 years	15	6	9	0.1768
≥ 50 years	24	10	14	
Tumor Size				
< 2 cm	15	7	8	0.0191^*^
≥ 2 cm	24	9	15	
Lymph node metastasis				
Yes	28	9	19	0.0273^*^
No	11	7	4	
TNM stage				
I-II	13	7	6	0.0301^*^
III-IV	26	9	17	
Distant metastases				
Yes	23	8	15	0.0986^*^
No	16	8	8	
ER status				
Positive	19	10	9	0.2285
Negative	20	6	14	
PR status				
Positive	25	12	13	0.4926
Negative	14	4	10	
HER-2 status				
Positive	12	5	7	0.1780
Negative	27	11	16	
Molecular subtype				
Luminal like	20	9	11	0.3483
HER-2 positive	11	5	6	
Triple negative	8	2	6	
Histological differentiation				
Well	11	4	7	0.6854
Moderate	19	7	12	
Poor	9	5	4	

*ER* estrogen receptor, *PR* progestrone receptor, *HER-2* human epidermal growth factor receptor-2, *TNM* Clinicopathologic stage

^*^ presents the statistic difference less than 0.05

### LncRNA LINC00511 promotes malignant cell proliferation and invasion in vitro and vivo

**Fig. 2:**
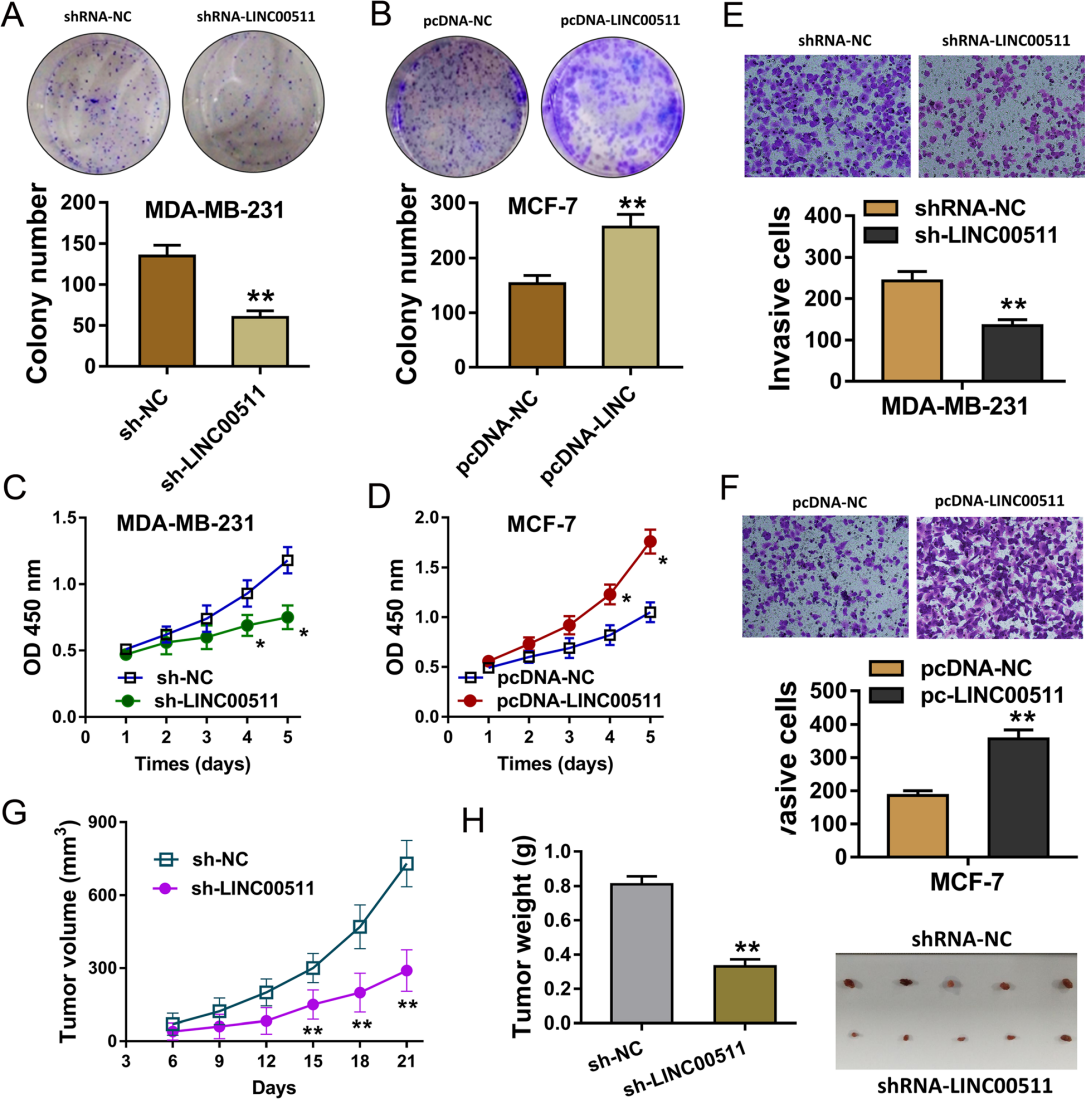
LncRNA LINC00511 promotes cell proliferation and invasion in vitro and vivo. **a**, **b** Colony formation assay in MDA-MB-231 and MCF-7 cells transfected with sh-LINC00511 or sh-NC. **c**, **d** CCK-8 assay presented the proliferation of MDA-MB-231 and MCF-7 cells transfected with sh-LINC00511 or sh-NC. **e**, **f** Number of invaded MDA-MB-2 31 cells and MCF-7 cells. **g**, **h** Tumor growth (volume and weight) in vivo. ** indicates *p* value less than 0.01. * indicates *p* value less than 0.05

Colony formation assay and CCK-8 assay revealed that LINC00511 silencing inhibited the proliferation of MDA-MB-231 cells, while enhanced LINC00511 expression promoted the proliferation of MDA-MB-231 cells (Fig. 2a, b, c, d). The transwell invasion assay analysis found that LINC00511 silencing reduced the number of invaded MDA-MB-231 cells, while enhanced LINC00511 expression produced the opposite effect (Fig. 2e, f). Finally, xenograft model in mice assay showed that LINC00511 silencing inhibited the tumor growth (volume and weight) in vivo (Fig. 2g, h). Overall, results found that lncRNA LINC00511 promotes the proliferation and invasion of malignant cells both in vitro and vivo.

### LncRNA LINC00511 contributes to the maintenance of breast cancer CSC characteristic

**Fig. 3:**
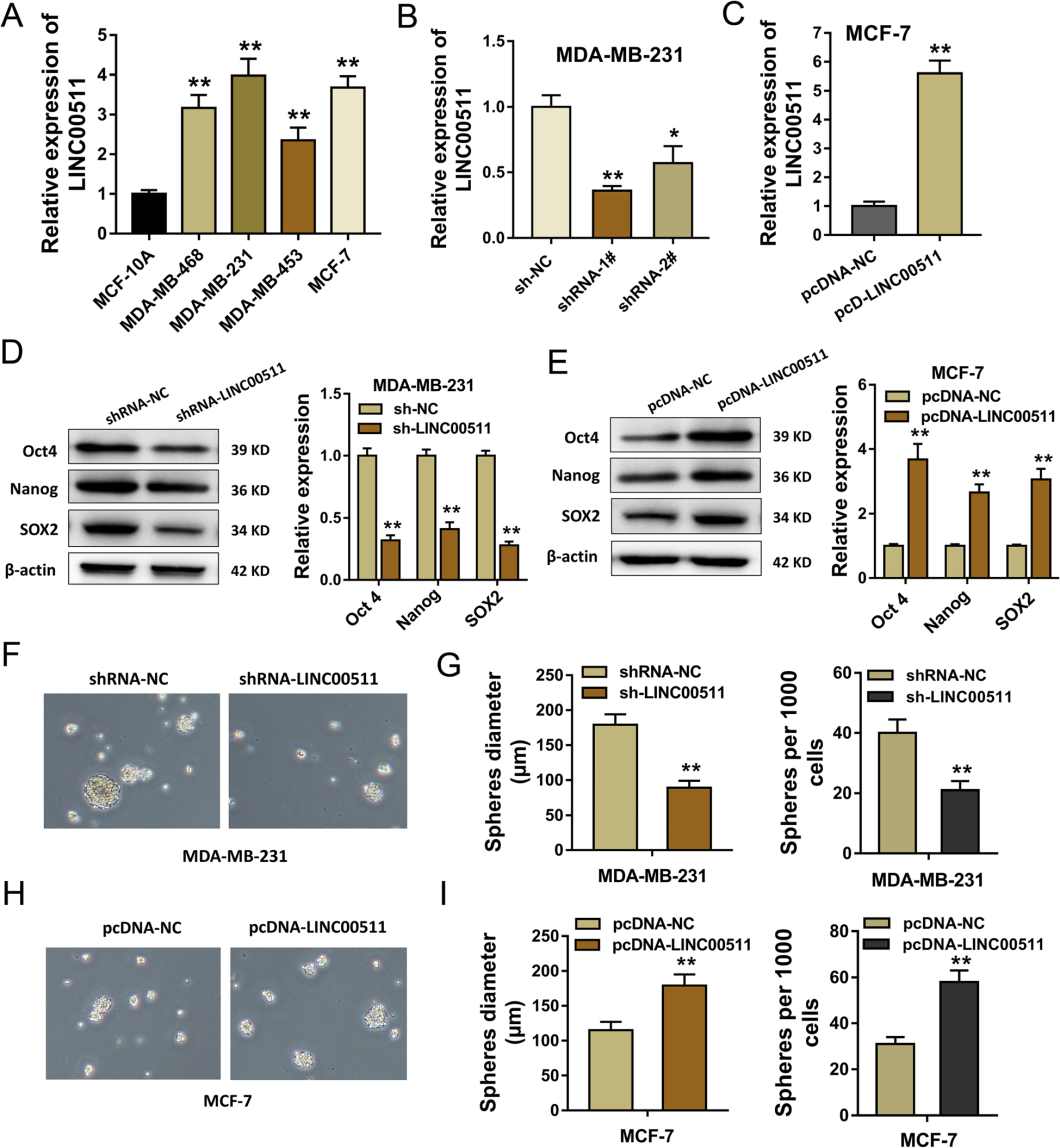
LncRNA LINC00511 contributes to the maintenance of breast cancer CSC characteristic. **a** LINC00511 expression in breast cancer cell lines MDA-MB-468, MDA-MB-231, MDA-MB-453, and MCF-7 and normal cell lines (MCF-10A). **b**, **c** Breast cancer cell MDA-MB-231, and MCF-7 LINC00511 expression. **d** Stem factor (Oct4, Nanog, SOX2) expression in MDA-MB-231 cells transfected with shRNA for LINC00511. **e** The stem factors (Oct4, Nanog, SOX2) expression in MCF-7 cells transfected with enhanced LINC00511 plasmids. **f**, **g** Mammosphere diameter and quantity when transfected with shRNA for LINC00511. **h**, **i** Mammosphere diameter and quantity when transfected with enhanced LINC00511 plasmids. ** indicates *p* value less than 0.01. * indicates *p* value less than 0.05

RT-qPCR found that LINC00511 expression was over-expressed in the breast cancer cell lines (MDA-MB-468, MDA-MB-231, MDA-MB-453, MCF-7) compared to control lines (MCF-10A) (Fig. 3a). In the functional cellular experiments, shRNA targeting LINC00511 and enhanced expression plasmids were transfected into breast cancer cells MDA-MB-231, MCF-7 to knockdown or enhance expression (Fig. 3b, c). Western blot analysis revealed that LINC00511 silencing suppressed stem factor (Oct4, Nanog, SOX2) expression in MDA-MB-231 cells (Fig. 3d), while enhanced LINC00511 expression significantly increased stem factor expression in MCF-7 cells (Fig. 3e). Sphere-formation assay showed that LINC00511 silencing reduced mammosphere diameter and quantity (Fig. 3f, g), while enhanced LINC00511 expression significantly increased mammosphere diameter and quantity (Fig. 3h, i). Results show that LINC00511 contributes to the maintenance of breast cancer CSC characteristics, indicating the role of LINC00511 in breast cancer cell stemness.

### LINC00511 targeted the miR-185-3p/E2F1 in the breast cancer cells as competing endogenous RNA

**Fig. 4:**
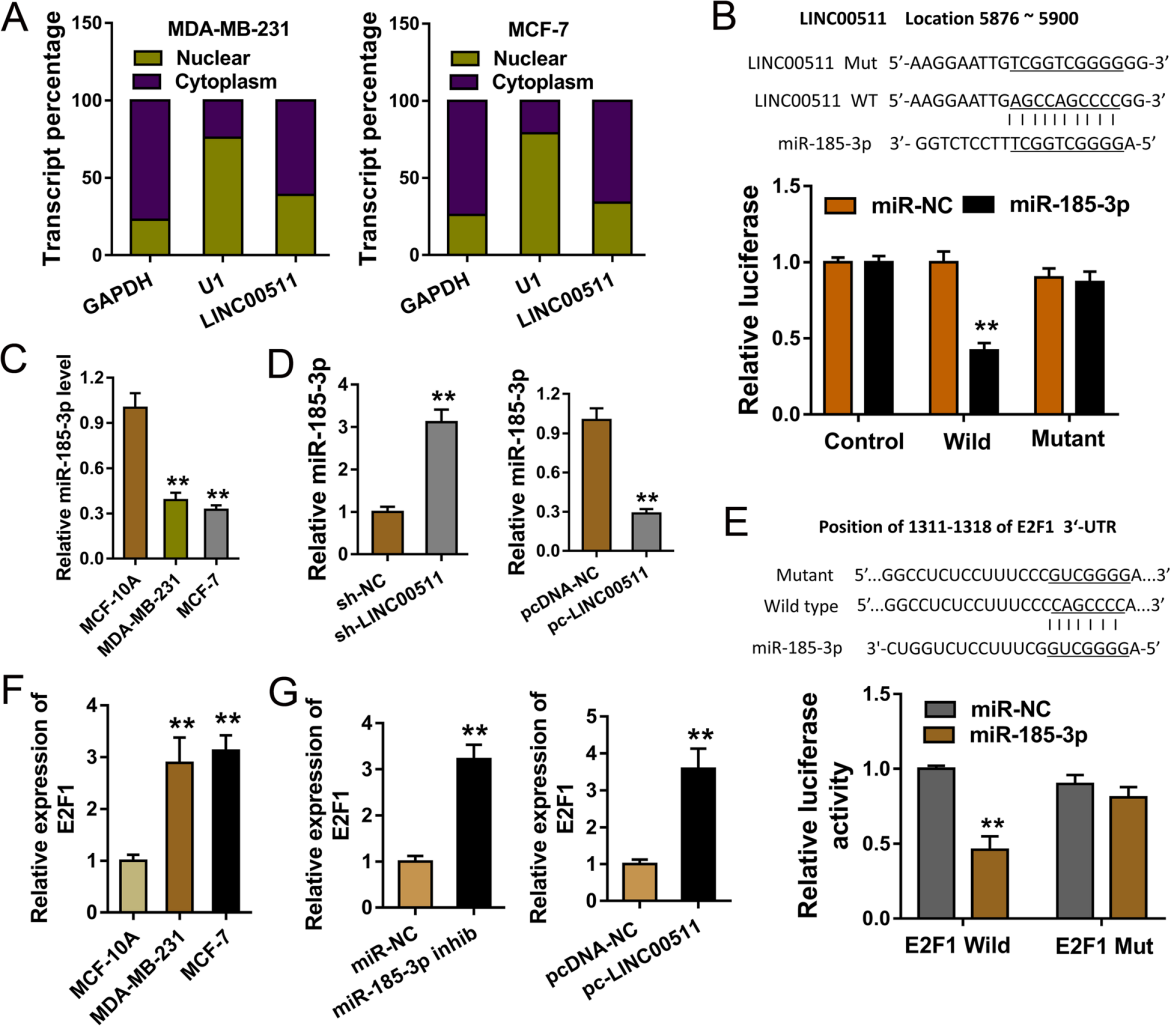
LINC00511 targeted the miR-185-3p/E2F1 in the breast cancer cells as competing endogenous RNA. **a** The subcellular position of LINC00511 on the cytoplasm or nucleus. GAPDH and U1 acted as the cytoplasm and nuclear control. **b** The binding sites of miR-185-3p and LINC00511. **c** miR-185-3p expression in the breast cancer cells MDA-MB-231, and MCF-7 measured by RT-PCR. **d** miR-185-3p expression in the transfection of LINC00511 silencing and enhanced LINC00511 plasmid. **e** miR-185-3p and E2F1 3’-UTR binding sites. **f** E2F1 mRNA expression in the breast cancer cells MDA-MB-231, and MCF-7 measured by RT-PCR. **g** E2F1 mRNA expression in the miR-185-3p inhibitor (miR-185-3p inhib) transfection and enhanced LINC00511 plasmid transfection. ** indicates *p* value less than 0.01. * indicates *p* value less than 0.05

Mechanical analysis revealed that LINC00511 is positioned on the cytoplasm rather than the nulceus in breast cancer cells MDA-MB-231, MCF-7 (Fig. 4a). This finding suggests that LINC00511 exerts its downstream effects by post-transcriptional epigenetic regulation. Results confirmed that LINC00511 had several potential binding sites with the downstream miRNAs (miR-185-3p), and the luciferase reporter assays found a strong relationship between miR-185-3p and LINC00511 (Fig. 4b). The expression of miR-185-3p was decreased in breast cancer cells MDA-MB-231, and MCF-7 (Fig. 4c). Moreover, miR-185-3p was over-expressed in the LINC00511 silenced transfection, while decreased in the enhanced LINC00511 plasmid transfection (Fig. 4d). Results also confirmed that E2F1 acted as the target of miR-185-3p, acting as a functional protein of miR-185-3p, which was further validated by luciferase reporter assays (Fig. 4e). Similarly, we found that E2F1 mRNA expression was increased in breast cancer cells MDA-MB-231, and MCF-7 (Fig. 4f) and up-regulated in the miR-185-3p inhibitor transfection and enhanced LINC00511 plasmid transfection (Fig. 4g). These results suggest that LINC00511 targets the miR-185-3p/E2F1 in breast cancer cells as ceRNA.

### E2F1 enhanced the Nanog expression at the transcriptional level

**Fig. 5:**
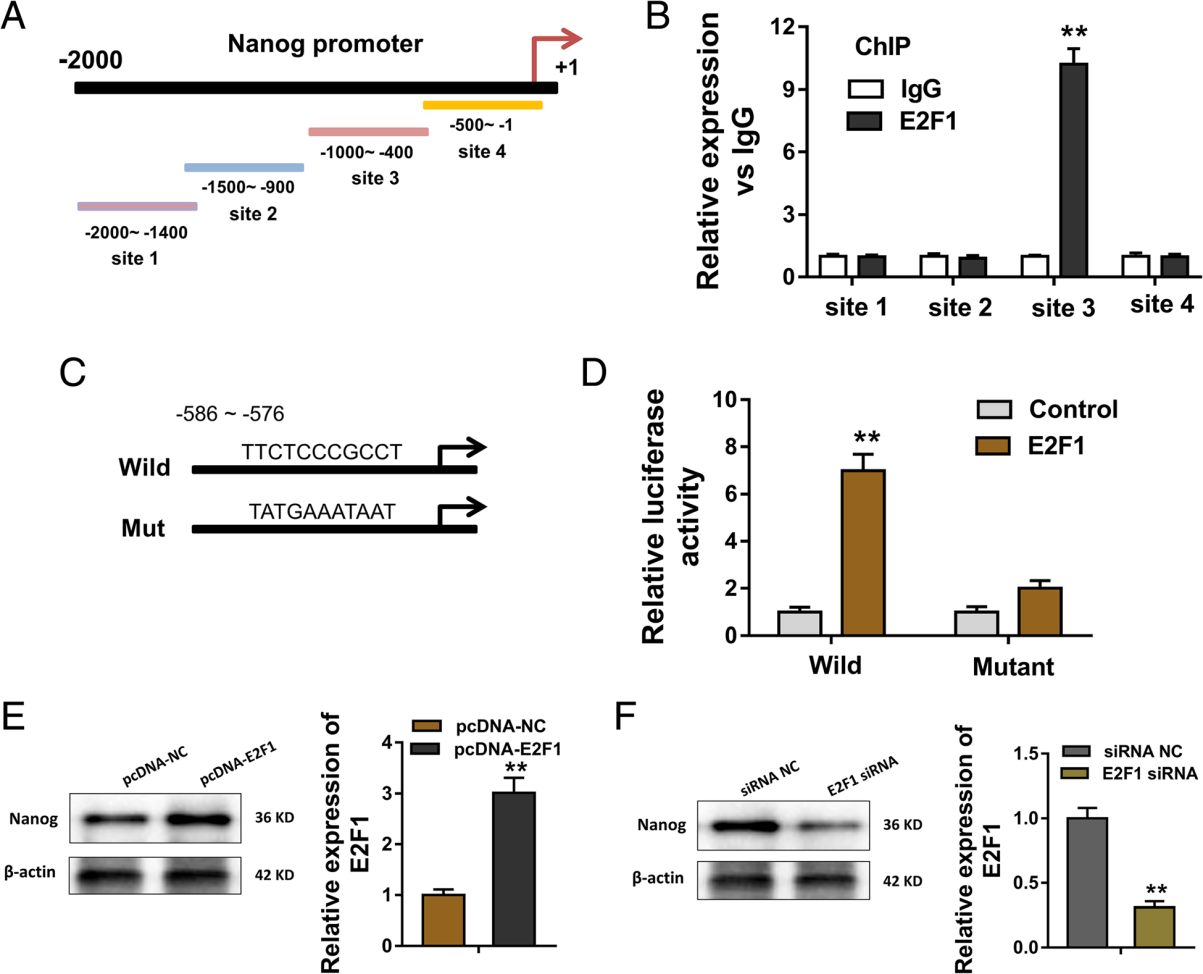
E2F1 enhanced Nanog expression. **a** The Nanog gene promoter region. **b** Chromatin immunoprecipitation (ChIP) assay was performed to identify which region functioned as the effective binding site of Nanog promoter region. **c** The luciferase reporter plasmid having wild type or mutant sequences of Nanog promoter region (− 586 ~ − 576) were established. **d** The luciferase activity of wild type sequences and mutant of Nanog promoter region. **e**, **f** Nanog protein expression when transfected with enhanced E2F1 plasmid or E2F1 silencing

**Fig. 6:**
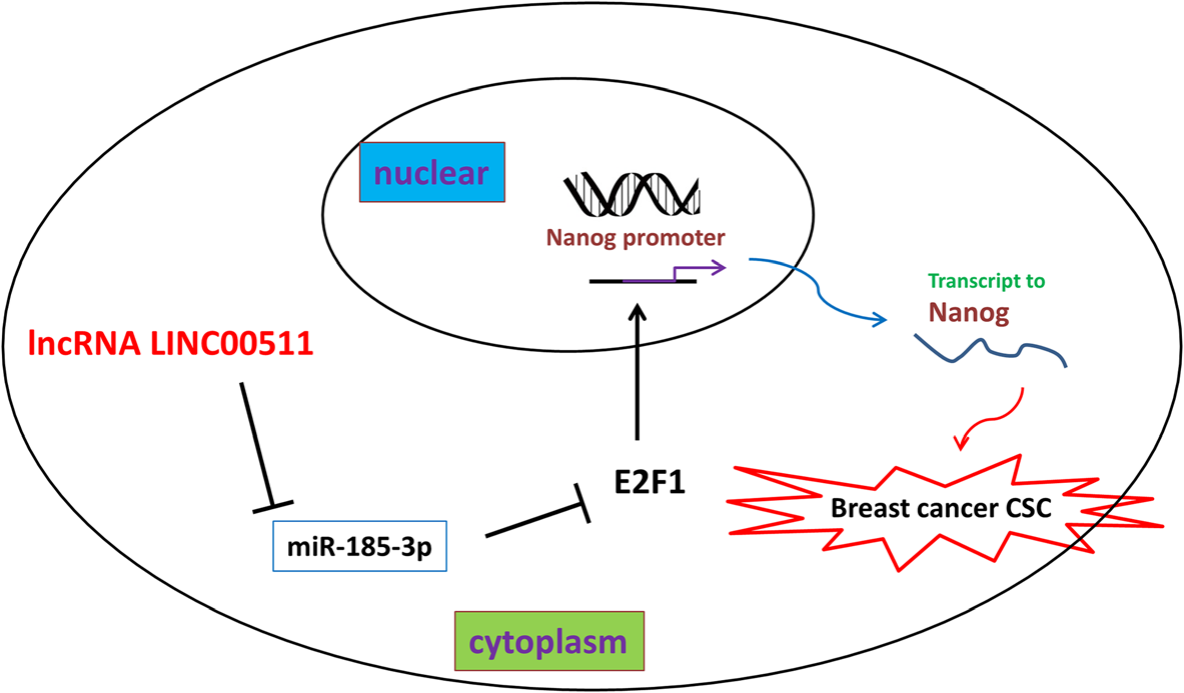
The mechanism by which LINC0051/miR-185-3p/E2F1 axis promoted breast cancer stemness and tumorigenesis

The promoter region of Nanog gene was divided into four sections and shown in Fig. 5a. Chromatin immunoprecipitation (ChIP) assay revealed that E2F1 endogenous binds with the (Additional file 3) Nanog promoter region sites (− 1000~ − 400) (Fig. 5b). Then, we performed bioinformatics tools to predict the binding sites of E2F1 for Nanog gene promoter. The bioinformatics tools are as following, UCSC (http://alggen.lsi.upc.es/cgi-bin/promo_v3/promo/), PROMO (http://alggen.lsi.upc.es/cgi-bin/promo_v3/promo/), JASPAR (http://jaspar.genereg.net/). In the promoter region of Nanog gene (+ 100~ − 2000 nt), we found that transcription factor E2F1 could bind with the region (− 586~ − 576) using PROMO and JASPAR. The wild type or mutant sequences of Nanog promoter region (− 586 ~ − 576) were established (Fig. 5c). Results indicated that the luciferase activity of wild type sequences of Nanog promoter regions was increased, while the mutant type was not. This suggests the binding site is within E2F1 and Nanog promoter region (Fig. 5d). Western blot indicted that enhanced E2F1 plasmid transfection increased Nanog protein expression (Fig. 5e), while the E2F1 silencing decreased Nanog protein expression (Fig. 5f). Results show that E2F1 enhanced Nanog expression at the transcriptional level, providing a potential mechanism by which LINC0051/miR-185-3p/E2F1 axis promotes breast cancer stemness and tumorigenesis (Fig. 6).

## Discussion

Breast cancer is a one of the most common cancers in women worldwide [20, 21]. Although treatment for breast cancer has improved, it still represents a high disease morbisity and mortality. Epigenetic modification is as a significant regulatory mechanism for human pathogenesis [22] and long noncoding RNAs (lncRNAs) have been found to play a vital role.

In the tumourigenesis of breast cancer, the stem properties and self-renewal capability can trigger the recurrence and metastasis of breast cancer cells [23–25]. The role of lncRNAs on cancer cell stemness have been widely reported in human cancers. For example, SOX2OT is up-regulated in glioma tissue and glioblastoma stem cells, and knockdown of SOX2OT inhibits the proliferation, migration and invasion of cells, and the SOX2OT-miR-194-5p/miR-122-SOX3-TDGF-1 pathway forms a positive feedback loop for glioma cell stemness [26]. High levels of OIP5-AS1 can post-transcriptionally modulate downstream target genes with promoter binding motifs and are activated by stemness-associated transcription factors in cancer [27].

In the present study, results found that lncRNA LINC00511 expression was increased in breast cancer tissue samples and cell lines and may be related to poor prognosis. These results suggest that LINC00511 may be an oncogene for breast cancer. The role of LINC00511 on the functional phenotype and stemness of breast cancer cells was investigated. Results of gain and loss-of-functional assays revealed that LINC00511 promotes sphere-formation, stem factor (Oct4, Nanog, SOX2) expression, and contributes to the maintenance of breast cancer CSC characteristic, indicating the role of LINC00511 in breast cancer cell stemness. Finally, results found that LINC00511 promotes cell proliferation and invasion in vitro and vivo [28, 29].

Up to now, CSCs have been isolated from multiple tumors, such as breast cancer, lung adenocarcinoma, colorectal cancer, malignant melanoma, and glioma [30]. The invasion and metastasis of tumor is an important malignant biological phenotype of tumor. Recently, increasing studies have suggested that CSCs play a vital role in tumor invasion and metastasis [31].

Further experiments investigated the mechanism by which LINC00511 regulates breast cancer pathophysiology. Results found that LINC00511 functions as a miR-185-3p ‘sponge’ to harbor its expression, and then target E2F1 protein, confirming the role of LINC00511/miR-185-3p/E2F1 axis in breast cancer. Similar results have found that lncRNA H19 correlates positively with LIN28 by acting as a ceRNA for miRNA let-7, to form a double-negative feedback loop, thereby regulating the maintenance of breast cancer CSCs [32].

Finally, results show that E2F1 acts as a transcription factor that binds with the Nanog promoter region. Luciferase reporter assay, ChIP and western blot confirmed the direct action within E2F1 and Nanog, enriching the regulatory pathway to be LINC00511/miR-185-3p/E2F1/Nanog. The transcription factor E2F1 has been identified to be a oncogenic element in bladder cancer and colorectal cancer [33, 34]. E2F1 was also reported to up-regulated and involved in the carcinogenesis of breast cancer and results found it is involved in the expression of Nanog in breast cancer.

As regarding to the role of lncRNAs on the breast cancer CSCs properties, we discover the vital pathway of LINC00511/miR-185-3p/E2F1/Nanog. Moreover, emerging evidence and published papers also reveal the relevant findings. For example, lncRNA HOTAIR tightly regulates the proliferation, colony formation, migration and self-renewal capacity of breast cancer CSCs, and specifically inhibits miR-34a, which leads to the upregulation of Sox2 protein [35]. Thus, it is clear that lncRNAs could powerfully regulate the breast cancer stemness and tumorigenesis [36].

## Conclusion

Results from this study show that LINC00511 acts as an oncogenic RNA in breast cancer tumorigenesis when present at high levels. LINC00511 functions as a miR-185-3p ‘sponge’ to harbor its expression, and then target E2F1 protein, which binds with the Nanog promoter region to activate its transcription. The characterization of the LINC00511/miR-185-3p/E2F1/Nanog axis provides an important insight for breast cancer stemness and tumorigenesis.

## Additional files

Additional file 1: **Table S1**. Sequences of shRNA and qRT-PCR. (DOCX 17 kb)

Additional file 2: **Table S2**. Primers sequences for ChIP. (DOCX 16 kb)

Additional file 3: **Table S3.** Nanog promoter region. (DOCX 16 kb)

## Abbreviations

ceRNA: Competing endogenous RNA
ChIP: Chromatin immunoprecipitation
GAPDH: Glyceraldehyde-3-phosphate dehydrogenase
lncRNA: Long non-coding RNA
PCR: Polymerase chain reaction
RIP: RNA immunoprecipitation
