# Supplementary material for: Long noncoding RNA LINC00511 contributes to breast cancer tumourigenesis and stemness by inducing the miR-185-3p/E2F1/Nanog axis
Source: J Exp Clin Cancer Res. 2018 Nov 27;37:289. doi: 10.1186/s13046-018-0945-6 (PMC6260744; doi:10.1186/s13046-018-0945-6)
Supplement: Supplementary file 1 — Table S1. Sequences of shRNA and qRT-PCR. (DOCX 17 kb) [file 13046_2018_945_MOESM1_ESM.docx]

**Table S1**. Sequences of shRNA and qRT-PCR.

|  | 5’-3’:forward and reverse |
| --- | --- |
| LINC00511 | forward, 5’-CTAACAAGAGGGTAAGTGTCAG-3'  reverse, 5'-AAGTCGACAACCCCATCGTTAC-3’ |
| miR-185-3p | forward, 5’-GATCACACTCTTGTGGTAGTTGC-3’  reverse, 5’-CTCTTCCTTGCTCGTTGTTGGTAT-3’ |
| E2F1 | forward, 5’-TGATTGTGGCAAAGGAGGA-3’  reverse, 5’-TTGGGTCATCATCACAGACG-3’ |
| GAPDH | forward, 5’-CTAAGGCCAACCGTGAAAAG-3’  reverse, 5’-ACCAGAGGCATACAGGGACA-3’ |
| sh-LINC00511-1 | 5’-GTGCAGTTCAACTAATAAATT-3’ |
| sh-LINC00511-2 | 5’-GCCAGAGTTGAATACCCTCA-3’ |
| miR-185-3p inhib | 5’-ACGGAUGGGAUUCCAAACUGGAC-3’ |
