# Supplementary material for: Long noncoding RNA LINC00511 contributes to breast cancer tumourigenesis and stemness by inducing the miR-185-3p/E2F1/Nanog axis
Source: J Exp Clin Cancer Res. 2018 Nov 27;37:289. doi: 10.1186/s13046-018-0945-6 (PMC6260744; doi:10.1186/s13046-018-0945-6)
Supplement: Supplementary file 2 — Table S2. Primers sequences for ChIP. (DOCX 16 kb) [file 13046_2018_945_MOESM2_ESM.docx]

**Table S2**. Primers sequences for ChIP.

|  | Sequences 5’-3’ |
| --- | --- |
| Site 1 | forward, 5’-ACCTTCCGCCTGACACCTTTGC-3’  reverse, 5’-TCGGCGGCCTCAACAATGG-3’ |
| Site 2 | forward, 5’-TAGATCAGAATAGTCAATGGTGGA-3’  reverse, 5’-AACAAATGTTTTAGCATTGGGATCT-3’ |
| Site 3 | forward, 5’-TGTGGCAGAAAGGATTGGA-3’  reverse, 5’-TTGCAGGGTCATCATCAACG-3’ |
| Site 4 | forward, 5’-CTCGTACCAGGCGAAAAAAG-3’  reverse, 5’-ACCAGAGGGGACACAGTACA-3’ |
