# Supplementary material for: Long noncoding RNA LINC00511 contributes to breast cancer tumourigenesis and stemness by inducing the miR-185-3p/E2F1/Nanog axis
Source: J Exp Clin Cancer Res. 2018 Nov 27;37:289. doi: 10.1186/s13046-018-0945-6 (PMC6260744; doi:10.1186/s13046-018-0945-6)
Supplement: Supplementary file 3 — Table S3. Nanog promoter region. (DOCX 16 kb) [file 13046_2018_945_MOESM3_ESM.docx]

The promoter region of Nanog gene (-2000~0)

Transcription factor E2F1 binds with the sites of Nanog gene promoter at -586~-576 (TCTCCCGC).

>hg19_knownGene_uc009zfy.1 range=chr12:7939995-7948755 5'pad=0 3'pad=0 strand=+ repeatMasking=none

CGCAAAGTGCTGGGATTACAGGTGTGAGCCACCGTGCCCAGCCGTTAGCT -2000

CATTTTAACACATCCTTAGTCCAGCCTGTTCCAAAAAATCTAAAGTCAGA

TAGCTTCCTAAACCTCAACTTTATTCCAATTGCTTTCCTTGGCGAAGAAT -1900

GTAGTAAGTCGGCCTTCCAGCCACCAGCCCCTTCCCTTTGGTCTTTCACT

CCGGAGGCTCTTACCCTAGACACAATGGGACAGGGAGCGGGGGATGGGGG -1800

AATTCAGCTCAGGCTTTTATGCAAAGACCCCCTTCTGCAAAGAACAAAGC

TTCTGGTACCTGCCCTTTGGAGAGCTGCGGGCAAGCTCAGCCTCGGTGAG -1700

TCTTGGTGGCCTTGACAGCCCCCACTTAACAAACTGTGCTGATTAAGAGA

GACAGGAGGGCAAGTTTTTCCCTTCTTTTAAAGAAATCATCCTATTTCCT -1600

ACGAGACATAGACTATCTGCCTGAAGCATGATGTACTAGCCCCACTCACC

GGCTCCCTGATGCCCCTATGCTTAATCTTCTCCGGAATGGTAGTCTGAGA -1500

AGAAAAAAGATTACGCCCAATTTCATTTCCTTGTTTCACATCAAGCAATA

CTTTTCGAGTCTTTGCATTGTGAACAAAAGTCAGCTTGTGTGGGAGCAAA -1400

GCCAGCTGCTCTGGGTGCAGACCCAGGAGCAGAGTGCAGAGGAGAATGAG

TCAAAGAGTTTTGTCTTCAAAAATTACATAATCGGGATTTGCTAAGAGTT -1300

TACTTTTCGGTATGGAAGACTGGAAAAGAGAAAGAAATCTTAGGTTTCTT

GAATGTTGGGTTTGGGAATAGGAAGGAAAATCGAAAACTGTAGACTTTGT -1200

CCATAAATGTTAGTGCTGGAACCCCACTCTAAAAACTTTGTTCCTTTGGA

AAACACCTCCCTTCCCCCAGAAACACACACACCCACACGAGATGGGCACG -1100

GAGTAGTCTTGAAAGACATGACAAATCACCAGACCTGGGAAGAAGCTAAA

GAGCCAGAGGGAAAAAGCCAGAAGTCGACTACCTGGGAGGAGGGATAGAC -1000

AAGAAACCAAACTAAAGGAAACTAAGGTAGGTGCTGAAAACAAGTACCAT

TTTCAACATTAACTGATGCCTTGGCTTCATGCTATAATGCCATGTTGTGT -900

TTCACTATAACCTCAGAGTGAATGAAAGAGGAAAATGGAGCTAGTTGAAA

TTTCTGCCTAAACTAGCCAGATTTTGAGACACTAAGTTATCTCAAATCAA -800

GAAATCACCCTAATGAGAATTTCAATAACCTCAGGAATTTAAGGTGCATG

CATCCCCCACCCCCCCCTTTTTTTTTTGAGACGTAGTCCCGCTCTGTTGC -700

CCAGGCTGGAGTACAGTGGCGCGATATCGGCTCACCACAACCTCTGCCTC

CCAGGTTCAAGGGATTCTCCCGCCTCAGCTTCCAGAGTAGCTGGGACTAC -600 ~ -551

AGACACCCACCACCATGCGTGGCTAATTTTTGTATTTTTAGTAGAGAGGG

GGTTTCGCCATGTTGGCCAGGCTGGTTTCAAACTCCTGACTTCAGGTGAT -500

CCGCCTGCCACGGCCTCCCAATTTACTGGGATTACAGGGGTGGGCCACCG

CGCCCGGCCTTTTTCTTAATTTTTAAAAATATTAAAGTTTTATCCCATTC -400

CTGTTGAACCATATTCCTGATTTAAAAGTTGGAAACGTGGTGAACCTAGA

AGTATTTGTTGCTGGGTTTGTCTTCAGGTTCTGTTGCTCGGTTTTCTAGT -300

TCCCCACCTAGTCTGGGTTACTCTGCAGCTACTTTTGCATTACAATGGCC

TTGGTGAGACTGGTAGACGGGATTAACTGAGAATTCACAAGGGTGGGTCA -200

GTAGGGGGTGTGCCCGCCAGGAGGGGTGGGTCTAAGGTGATAGAGCCTTC

AAATCTTTGTTAAATTTTTGGTTGGGGTGGAGAAGGAAATTAGCTGAGGA -100

CACTGCTATCTTAGAAATGCATAGAAATAGCTGAGCGTGGTGGCCTATGC

-------------------------------------------------------------------------------------- 0
